# An evaluation of the utility of routine laboratory monitoring of juvenile idiopathic arthritis (JIA) patients using non-steroidal anti-inflammatory drugs (NSAIDs): a retrospective review

**DOI:** 10.1186/1546-0096-8-11

**Published:** 2010-04-14

**Authors:** Sheetal S Vora, Christine E Bengtson, Grant D Syverson, James J Nocton

**Affiliations:** 1 Department of Pediatrics, Division of Rheumatology, Medical College of Wisconsin, Children's Research Institute, Milwaukee, WI, USA

## Abstract

**Background:**

No consensus evidence-based guidelines for the routine laboratory monitoring of children with JIA receiving non-steroidal anti-inflammatory drugs (NSAIDs) exist. The purpose of this study is to evaluate the clinical utility of routine laboratory monitoring of hemoglobin, transaminases, blood urea nitrogen, serum creatinine, and urinalysis in patients with juvenile idiopathic arthritis (JIA) receiving NSAIDs.

**Methods:**

The medical records of 91 children with JIA followed between 1996 and 2006 were retrospectively reviewed for laboratory results and clinically significant adverse effects attributed to NSAID use. Laboratory abnormalities were documented, with potential adverse clinical sequelae, including if NSAID use was discontinued.

**Results:**

Abnormal laboratory results were recorded for 24 of 91 patients. Nearly all abnormalities were mild and not associated with adverse clinical sequelae. All patients but one continued to receive NSAID therapy after the abnormality was detected.

**Conclusions:**

Although detection of abnormal laboratory values occurred while on NSAIDs, these abnormalities did not correlate with adverse clinical signs and symptoms. The routine monitoring of laboratory tests in asymptomatic children treated with NSAIDs is of questionable utility.

## Background

Juvenile idiopathic arthritis (JIA) continues to be the most common chronic rheumatic disease affecting children worldwide[1]. Optimal clinical management of JIA is multidisciplinary and includes medication, physical and occupational therapy, psychosocial support services, and frequent monitoring of both disease activity and potential adverse effects of treatment[2,3]. Non-steroidal anti-inflammatory drugs (NSAIDs) are a mainstay of treatment for almost all children with JIA at some stage of their disease[4]. NSAIDs inhibit cyclo-oxygenase (COX), decreasing the production of pro-inflammatory prostaglandins. Potential adverse effects of NSAIDs include dermatitis, gastritis, hepatitis, and nephritis. These adverse effects appear to be less common in children [4-6], although the true incidence is unknown.

While the incidence of clinically associated adverse effects due to NSAIDs appears to be relatively low in children with JIA [4-6], many pediatric rheumatologists continue to perform routine laboratory monitoring in children receiving chronic NSAID therapy. The American College of Rheumatology has developed NSAID laboratory monitoring guidelines for adults with rheumatoid arthritis [7], but these guidelines may not be appropriate for children.

We reviewed the charts of JIA patients receiving NSAIDs followed at the Children's Hospital of Wisconsin to determine the frequency of abnormal serum transaminases, blood urea nitrogen (BUN), serum creatinine (Cr), hematuria, proteinuria, and hemoglobin (Hgb), and the clinical outcome of those with an abnormality, including any potential dermatitis, gastritis, hepatitis, and nephritis. Our aim was to estimate the frequency of significant adverse clinical events associated with or attributed to NSAID toxicity that would be detected by routine laboratory monitoring in children. This data may be useful in guiding clinicians on the utility of routine laboratory testing for children with JIA receiving NSAIDs.

## Methods

We retrospectively reviewed the charts and laboratory data of all children diagnosed with JIA and were followed in the pediatric rheumatology clinic at the Children's Hospital of Wisconsin between January 1996 and September 2006. All children met diagnostic criteria for JIA[8,9]. Inclusion criteria included treatment with NSAIDs for at least one month and a reported result on at least one occasion of a minimum of one of the following laboratory tests: aspartate aminotransferase (AST), alanine aminotransferase (ALT), BUN, serum Cr, urinalysis, or Hgb. Exclusion criteria included children with systemic-onset JIA. In children treated with methotrexate (MTX), laboratory results and clinical information were recorded only during the time interval prior to the initiation of MTX.

Laboratory data were reviewed and all abnormal laboratory values were identified during the time period of the study. The medical records of those with abnormal laboratory values were reviewed further and any associated adverse clinical event was recorded. For the purpose of this analysis, the discontinuation of NSAIDs was considered an adverse clinical event. Abnormal values of laboratory tests were defined by the laboratory standards of Children's Hospital of Wisconsin. In addition, patient age, gender, subtype of JIA, the frequency of laboratory monitoring, the median time period while receiving NSAIDs, the type of NSAID prescribed, and any additional medications prescribed were recorded for all patients.

## Results

91 patients met the inclusion criteria and were included in the data analysis. Of these, 62 (68%) had oligoarticular disease and 29 (32%) had polyarticular disease (Table 1). Seventy-four (81%) were female and 17 (19%) male. The frequency of laboratory monitoring for individual patients varied significantly. Serum AST was tested a median of 2 times (range of 0-17) per patient, ALT 2 times per patient (range 0-14), and Hgb 2 times per patient (range 0-15), serum Cr 1 time per patient (range 0-10) and urinalysis 0 times per patient (range 0-9). Median duration of treatment with NSAIDs was 12 months (range 1.33-212.80). Naproxen was the most commonly used NSAID (95% of patients). Nearly all patients only received non-selective COX inhibiting NSAIDs. All children received a single NSAID at a time. Few children were receiving additional medications in combination with NSAIDs, including biologic agents. Total patient years of observation on NSAIDs for both groups were 227.6 years. Specifically, 180.6 years for the oligoarticular JIA subtype and 46.9 years for the polyarticular JIA subtype.

**Table 1:** Clinical characteristics of 91 children with JIA.

	Patients	
Characteristic	**No**.	%
Median age at onset (yr)	4 (0.1-15.7)	
Gender		
Female	74	81
Male	17	19
JIA onset subtype		
Oligoarticular	62	68
Polyarticular	29	32
NSAID*		
Naproxen	87	95
Meloxicam	4	4
Tolmetin	11	12
Ibuprofen	16	18
Rofecoxib	6	7
Diclofenac	8	9
Celecoxib	3	3
Nabumetone	2	2
Other Medications†	6	7
Median duration of treatment (yr)		
Oligoarticular	1.5 (0.14-17.7)	
Polyarticular	0.58 (0.11-9.8)	

* Some patients may have been treated with more than one NSAID

(NSAIDs were never used simultaneously)

† Azathioprine (1), Etanercept (1), Hydroxychloroquine (4)

Abnormal laboratory results were documented in 24/91 (26%) patients. Of those with oligoarticular disease, 5 had abnormally low hemoglobin (9.9-11.6), 7 had elevated serum transaminases (47-406), 9 had elevated BUN (21-24), and 1 had trace proteinuria. Five patients had abnormalities in two categories. In only one patient with abnormalities in both serum transaminases and hemoglobin was the NSAID discontinued (Table 2). In this patient, the cause for the elevated transaminases remained unclear even following liver biopsy. Of the 7 patients with polyarticular disease and detected laboratory abnormalities, 5 had abnormally low hemoglobin (8.4-10.6), 1 had elevated BUN (22), and one had trace proteinuria. None had more than one abnormality and none had an abnormality requiring discontinuation of NSAID therapy (Table 3). None of the 23 patients who continued to receive NSAIDs had a clinically significant adverse event that was felt to be secondary to the NSAID.

**Table 2:** Abnormalities of Laboratory Values in Oligoarticular JIA Patients.

Gender	Variable	Value	Observations	NSAID Continued
Female	BUN	22	1	Yes
Female	AST	47-48	2	Yes
Female	ALT	45	1	Yes
Female	BUN	22	1	Yes
Female	AST	46	1	Yes
	ALT	50-67	5	Yes
Female	AST	57-267	4	Yes
	ALT	67-406	4	Yes
	BUN	23	1	Yes
Female	BUN	21	1	Yes
	Proteinuria	Trace	1	Yes
Female	Hgb	9.9-10.1	5	Yes
Female	AST	49	1	No
	ALT	50-107	5	
	Hgb	11	1	
Female	BUN	21	1	Yes
Male	BUN	21	1	Yes
Female	AST	48-49	2	Yes
Female	BUN	21	1	Yes
Female	BUN	25	1	Yes
	Hgb	11	2	Yes
Female	Hgb	10.8	1	Yes
Male	ALT	44	1	Yes
	BUN	24	2	Yes
Female *	Hgb	11.6	1	Yes

* This patient was being treated with Tolmetin; all others were treated with Naproxen.

**Table 3:** Abnormalities in Polyarticular JIA Patients.

Gender	Variable	Value	Observations	NSAID Continued
Female	Proteinuria	Trace	1	Yes
Female	Hgb	8.4	1	Yes
Female	Hgb	10.1	1	Yes
Female	Hgb	10.4	1	Yes
Female	Hgb	10.2-10.6	3	Yes
Female	Hgb	9.5-10.5	5	Yes
Female	BUN	22	1	Yes

## Discussion

The results of this study suggest that the routine laboratory monitoring of children with JIA receiving NSAIDs alone has limited clinical utility. Although the frequency of results falling outside the normal range as determined by the laboratory was high (26%), most of these results were mildly abnormal and clinically insignificant. There was only one patient of the 91 included in our study in which laboratory monitoring detected an abnormality that led to discontinuation of the NSAID, and no patients had an adverse event clearly related to NSAIDs and detected by laboratory monitoring.

Our results are consistent with those of previous studies that have evaluated unselected cohorts of patients with Juvenile Rheumatoid Arthritis (JRA). In a prospective study evaluating potential renal complications of children with JRA receiving NSAIDs for 6 months or longer, 22 of 226 children (10%) had findings of microscopic hematuria and/or proteinuria on one or more urinalyses[5]. However, none of the children developed hypertension, and in 21 of the patients the abnormalities resolved spontaneously. In 1 patient, proteinuria continued 1 1/2 years later despite discontinuing NSAIDs, therefore the relationship to NSAID use remained unclear. A retrospective study of potential gastrointestinal adverse effects of NSAIDs in an unselected cohort of children with JRA concluded that the prevalence of clinically significant gastropathy associated with NSAID use in children with JRA was approximately 1%[4]. Of 702 children followed over 15 years, 10 events of clinical significance described as esophagitis, gastritis, or peptic ulcer disease were identified. The 10 events all occurred in symptomatic children and no adverse events were discovered by routine laboratory monitoring of asymptomatic patients [4].

Previous studies which have reported a wider range of adverse events potentially related to NSAID use, including some that are significantly higher, are limited by small sample sizes and potential selection bias. In a United Kingdom study of 13 children on NSAIDs with Juvenile Chronic Arthritis (JCA) complaining of abdominal pain or nausea, endoscopic findings were mild. Only one patient had a single duodenal erosion at first endoscopy to account for an unexplained drop in Hgb associated with mild nausea. They determined that significant ulceration commonly seen in adults was not observed and there was no correlation between the small number of petechial lesions and mild gastritis with reported symptoms [10]. Mulberg et al. reported endoscopically-documented gastric injury in 13 of 17 patients (76%) with JRA referred for epigastric abdominal pain, hematemesis, guiac positive stools and/or iron deficiency anemia [11]. These patients were evaluated prospectively, however only symptomatic patients were eligible. The evaluation included a complete blood cell count and evaluation of the stool for occult blood in all patients, followed by endoscopy. There was a strong correlation between gastric lesions and the presence of epigastric pain, regardless of the presence of anemia or occult blood in stool. Similarly, a retrospective study by Dowd et al. of children with arthritis receiving NSAIDs reported that 16 of 47 patients (34%) with symptoms of significant abdominal pain were found to have gastric or duodenal radiographic injury [12]. As with Mulberg's study, however, only patients with gastrointestinal symptoms were evaluated. Our study did not attempt to identify whether or not laboratory testing was performed as a result of patients having symptoms that might have been related to NSAID use. We instead chose to review all laboratory testing performed during this period, and identify all detected abnormalities. Although we have concluded that routine laboratory screening may have limited utility, laboratory testing of select symptomatic children may be of greater clinical value.

There are no consensus evidence-based guidelines for the routine laboratory monitoring of children with JIA receiving NSAIDs. Recommendations vary, but typically include monitoring serum liver and renal function along with a complete blood count (CBC) every 3 to 6 months [9,13]. The Committee on Clinical Guidelines for monitoring drug therapy in adults with rheumatoid arthritis recommends yearly CBC and liver function tests depending on type of NSAID [7]. The incidence of reported NSAID adverse events in adult patients ranges from rare (<1%) to uncommon (1-10%) to common (>10%) and states that insufficient data is available to develop complete evidence-based recommendations on the extent and frequency of monitoring. Furthermore, the guidelines also say it is unlikely that studies which gather such data will be performed as toxicities in adults range form 0.1% to 5% [7]. From this, it is expected that evidence is lacking to support the application of these guidelines to the care of children.

Our study has several limitations. As a result of the retrospective design, complete data was not available for all patients. Patients were not followed for a consistent period of time. The frequency of laboratory testing in general was relatively low and was inconsistent among practitioners and patients. There was no prior determination of either a consistent frequency of laboratory testing or the evaluation and management of children with abnormalities on testing. Specifically, there were no consistent criteria for discontinuing NSAID therapy. Since the discontinuation of NSAID was used as a surrogate for clinical significance, and since this study was undertaken in a single pediatric rheumatology clinic where the patients were cared for by a small number of practitioners, this determination may be susceptible to measurement bias. Sample size was also limited due to the lack of standardization regarding the frequency of testing as well as the exclusion of many children receiving methotrexate, who undergo routine laboratory monitoring specifically due to the potential for methotrexate to cause liver toxicity.

## Conclusions

This study was conducted to understand the frequency of abnormal laboratory tests associated with NSAID toxicity and then determine if those abnormalities led to clinically significant adverse events resulting in the discontinuation of NSAIDs. Our study suggests that clinically significant adverse events requiring the cessation of NSAIDs and detected by routine laboratory monitoring appear to be very infrequent. Therefore, frequent laboratory monitoring in patients with JIA receiving NSAIDs is unlikely to be cost effective. A prospective study to determine the incidence and prevalence of significant adverse effects detected by routine serial laboratory monitoring would be helpful in further defining the utility of routine laboratory testing in this population.

## List of abbreviations

NSAIDs: Non-steroidal anti-inflammatory drugs; JIA: Juvenile idiopathic arthritis; COX: cyclo-oxygenase; AST: aspartate aminotransferase; ALT: alanine aminotransferase; BUN: blood urea nitrogen. Cr: serum creatinine; Hgb: hemoglobin.

## Competing interests

The authors declare that they have no competing interests.

## Authors' contributions

SSV and JJN were responsible for study design. SSV, CEB, GDS acquired the data. SSV and CEB performed the statistical analysis and SSV and JJN interpreted the data. SSV, CEB, GDS and JJN were responsible for the manuscript preparation. All authors read and approved the final manuscript.
